# Sixth Annual Report of the Managers of the State Lunatic Asylum, Utica

**Published:** 1849-04

**Authors:** 


ART. III.—*Sixth Annual Report of the Managers of the State Lunatic Asylum, Utica. Made to the Legislature. February* 1, 1849.

The Annual Report of the Superintendent and Physician of the Utica Asylum, presents the following statistics:—

| | | |
|---|---|---|
| Remaining at the close of the last year, | | 472 |
| Admitted during year, | | 405 |
| | | 877 |
| Of this number, discharged | recovered, | 174 |
| " | improved, | 84 |
| " | unimproved, | 38 |
| | died, | 86 |
| | | 382 |
| Remaining at the Asylum, Nov. 30th, 1848, | | 495 |

The number of inmates during the last year was considerably larger than at any previous year since the institution was established.

During the months of August and September, severe dysentery and diarrhœa prevailed in the asylum, being prevalent, at the same time, in Utica and its vicinity. One hundred and ninety-five cases of dysentery, and twenty-seven cases of diarrhœa, occurred, making a total of two hundred and forty cases. Of this number, thirty-nine died—thirty-five of dysentery, and four of diarrhœa. The only son of Dr. Brigham, the superintendent, aged twelve years, a youth of great promise, fell a victim to the epidemic.

Dr. Brigham reiterates the advice given in his last annual report, not to transfer patients to the asylum too early after the development of insanity, particularly if the transfer requires a long journey. Mental derangement is frequently connected, at its incipiency, either with inflammation, or intense erethism of the encephalon, and the fatigue and excitement of removal, under these circumstances, is extremely prejudicial, often leading to fatal results. Medical men, who are, or should be consulted in all cases of insanity as to the propriety of removal, will do well to bear in mind this caution. A few weeks' delay in any case of recent insanity, is unattended by danger. In puerperal cases, five or six weeks should elapse after confinement, before the patient should be taken from home. Dr. B. states that patients with puerperal mania have been brought, from a distance, to the Asylum, *two weeks* after confinement! This, if done under the sanction of a medical practitioner, is quite inexcusable.

The report contains the following statistical facts relating to *hereditary predisposition to insanity:*—

| | | |
|---|---|---|
| Number of patients who have been at the Asylum, | | 2,014 |
| " | men, | 1,017 |
| " | women, | 997 |
| Known to have insane *relatives*, | | 637 |

| | | |
|---|---|---|
| Number of patients known to have insane *parents*, | | 273 |
| " | men, | 121 |
| " | women, | 152 |

| | |
|---|---|
| Of the 121 men, the number having insane fathers, was... | 64 |
| " " " " mothers, was... | 53 |
| Of the 152 women, the number having insane fathers, was | 67 |
| " " " " mothers, was | 80 |

And four men and five women inherited a predisposition to insanity from both parents.

Thus it would appear from our inquiries, and they have been very carefully conducted, that insanity is a little more likely to be transmitted by the mother than by the father, and that mothers are considered more likely to transmit it to daughters than to sons; while the fathers most frequently transmit it to sons.

Dr. Brigham submits a concise enumeration of his views respecting the *causes, prevention,* and *treatment* of insanity, to which we cannot do justice by any abridgement, and must therefore refer the reader to the Report itself, which, we hope, will be extensively circulated. He dwells with emphasis on the importance of a due amount of sleep in the prophylaxis, deeming morbid vigilance a condition which exerts a powerful agency in the production of insanity, when it concurs with a predisposition, or other causes. His remarks on this point seem to us to possess reasonableness and importance.

As regards the general management of the Asylum, we discover nothing in the present report calling for particular notice. The continued prosperity of the institution affords ample evidence of the ability and faithfulness of those to whom its management is entrusted.

BUFFALO, March, 1849.

